# Causal Effects Between Antibody-Mediated Immune Responses and Parkinson’s Disease: Insights from Genetic Studies

**DOI:** 10.7759/cureus.101998

**Published:** 2026-01-21

**Authors:** Fengyun Yu, Rong Cai

**Affiliations:** 1 School of Engineering Medicine, Beihang University, Beijing, CHN

**Keywords:** antibody-mediated immune responses, genetic association, mendelian randomization, parkinson' s disease, sensitivity

## Abstract

Background: Parkinson’s disease (PD) is a multifactorial neurodegenerative disorder with a complex etiology. Increasing evidence suggests that antibody-mediated immune responses elicited by prior infectious exposures may contribute to its pathogenesis; however, whether these associations reflect true causal relationships remains unresolved, largely due to confounding and other inherent limitations of observational study designs.

Methodology: We conducted a two-sample Mendelian randomization (MR) analysis to interrogate the causal associations between 46 antibody-mediated immune responses and PD risk, leveraging Genome-wide association study (GWAS) summary statistics for antibody-related traits and PD data from the FinnGen consortium (5,861 cases and 494,487 controls of European ancestry). Independent genetic variants reaching genome-wide significance were selected as instrumental variables. Causal estimates were primarily derived using the inverse-variance weighted approach, with complementary analyses conducted using the weighted median, MR-Egger regression, and both simple and weighted mode methods. Bayesian weighted MR, together with a series of sensitivity analyses, was employed to evaluate the robustness of the findings and interrogate potential heterogeneity and horizontal pleiotropy.

Results: Among the 46 antibody-mediated immune responses analyzed, two showed statistically significant causal associations with PD after Bonferroni correction. Genetically predicted elevated antibody levels against Epstein-Barr virus (EBV) nuclear antigen 1 (EBNA-1) were significantly associated with an increased risk of PD (odds ratio (OR) = 1.154, 95% confidence interval (CI): 1.098-1.214, *P* < 1.09 × 10^-4^). In contrast, genetically predicted seropositivity for anti-polyomavirus 2 IgG was associated with a reduced risk of PD ( OR = 0.897, 95% CI: 0.865-0.930, *P* < 1.09 × 10^-4^). Sensitivity analyses revealed no evidence of significant heterogeneity or horizontal pleiotropy.

Conclusions: This MR study offers genetic evidence supporting a potential causal involvement of specific antibody-mediated immune responses in the pathogenesis of PD. Elevated EBNA-1 antibody levels are associated with an increased risk of PD, whereas anti-polyomavirus 2 IgG seropositivity appears to confer a protective effect. These findings provide new insights into the immunological mechanisms underlying PD and underscore the potential of immune-related biomarkers and therapeutic targets.

## Introduction

Parkinson’s disease (PD) is a slowly progressive neurodegenerative disease characterized predominantly by motor dysfunction, including resting tremor and muscular rigidity. Collectively, these hallmark clinical features substantially compromise patients’ functional capacity and overall quality of life [1]. Against the backdrop of accelerating global population aging, the prevalence of PD has been increasing steadily. This trajectory not only leads to a marked deterioration in patients’ overall functioning but also places a considerable socioeconomic burden on affected families and on society at large. Large-scale meta-analyses indicate that approximately 6-7 million individuals worldwide are affected by PD, with a global incidence typically ranging from 20 to 30 cases per 100,000 population. Notably, the prevalence of PD in males is approximately 1.5-fold higher than in females. Projections further suggest that the total number of individuals living with PD will reach 25.2 million by 2050 [2].

The precise pathogenic mechanisms underlying PD remain incompletely understood. Current prevailing views suggest that PD pathogenesis may involve multiple factors, including aging, impaired protein clearance, and mitochondrial dysfunction [3]. Moreover, with the rapid expansion of knowledge regarding the pathological mechanisms underlying PD in recent years, an increasing body of evidence indicates that antibody-mediated immune responses directed against specific microorganisms may represent a key driver in the progression of PD pathology. As a central component of the mammalian adaptive immune system, antibodies play a crucial role in host defense against infection and in the establishment of long-term immunological memory. Investigating antibody-mediated immune responses constitutes a commonly adopted approach for probing the association between infectious exposures and the risk of non-communicable disorders. Seropositivity can play the role of a biomarker of prior viral exposure and may also provide pathophysiological insights into how infections contribute to the development of these disorders. For instance, a recent epidemiological investigation lent support to the multiple-microorganism hypothesis by showing that individuals seropositive for antibodies against a broad spectrum of pathogens, including herpes simplex virus type 1 (HSV-1), Epstein-Barr virus (EBV), Borrelia burgdorferi, Chlamydia pneumoniae, and Helicobacter pylori, had a significantly elevated risk of PD compared with healthy controls [4]. Moreover, the influenza A(H1N1) virus and the influenza A(H3N2) virus have all been associated with encephalitis lethargica and subsequent post-encephalitic Parkinsonism, a condition that is neuropathologically distinct from idiopathic PD [4]. However, all of the aforementioned evidence is derived from observational studies and may therefore be subject to confounding, reverse causality, and other sources of bias. Consequently, the causal relationship between specific antibody-mediated immune responses and PD remains unresolved. Accordingly, there is a critical need for the design and implementation of comprehensive, rigorously controlled studies to precisely delineate the relationship between immune responses and the risk of PD.

Mendelian randomization (MR) is a genetic epidemiological framework that leverages genetic variants, most commonly single-nucleotide polymorphisms (SNPs), as instrumental variables (IVs) to enable causal inference between putative exposures and disease outcome [5]. By exploiting the random assortment of alleles at conception, MR effectively mitigates confounding and reverse causality that commonly undermine conventional observational analyses [6]. Owing to its conceptual similarity to randomized controlled trials, MR has become a powerful tool for elucidating disease etiology in the era of large-scale genome-wide association studies. Consequently, we employed an MR framework to systematically examine the causal relationships between 46 antibody-mediated immune responses and susceptibility to PD.

## Materials and methods

Research strategy

**Figure 1:**
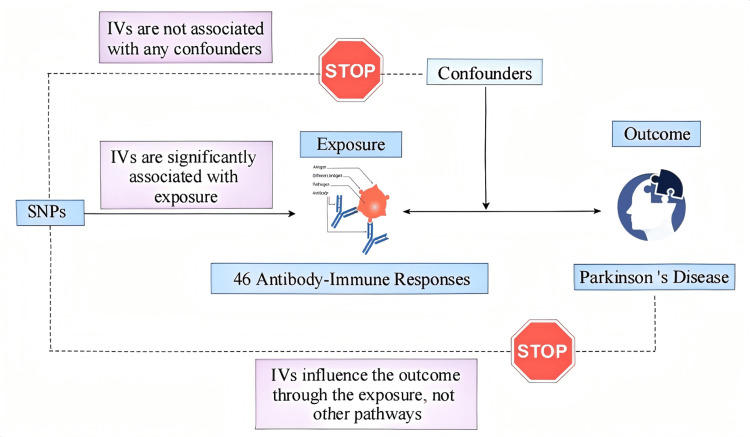
Flowchart of the MR analysis framework for 46 antibody-mediated immune responses and PD. The image elements were downloaded from BioRender (https://app.biorender.com) and then manually created using ProcessOn (https://www.processon.com). MR, Mendelian randomization; SNP, single-nucleotide polymorphism; PD, Parkinson’s disease; IV, instrumental variable

The research adopted a two sample MR design to assess the potential causal links between 46 antibody-mediated immune responses and the risk of PD. SNPs were used as IVs. As illustrated in Figure 1, valid IVs are required to fulfill three fundamental assumptions: they must be robustly linked to the exposure of interest, autonomous from latent confounders, and affect outcome exclusively through the exposure pathway [7].

Information sources

Genome-wide association study (GWAS) summary statistics for 46 antibody-mediated immune responses were derived from the work of Butler-Laporte et al [8]. To minimize the potential for sample overlap, we selected PD-related GWAS outcome data that were collected at different time points and from different geographic regions than the exposure datasets. PD data were derived from the FinnGen consortium, based on the GWAS dataset finn-G6-PARKINSON, which included 500,348 European participants (5,861 cases and 494,487 controls). Following stringent quality control procedures and genotype imputation, approximately 21.3 million SNPs were included in the analysis. In addition, all GWAS data used in this study were derived from populations of European ancestry. Given that all data were derived from publicly available resources, the study did not require additional ethical approval or the acquisition of informed consent [9].

Instrumental variable selection

SNPs significantly associated with 46 antibody-mediated immune responses were selected as IVs. Variants reaching genome-wide significance (*P *< 5 × 10^-5^) were considered candidate IVs. Linkage disequilibrium (LD) was controlled by applying a stringent *r*² threshold (*r*²<0.001) within a 10,000-kb genomic window [10]. Instrument strength was evaluated using the F statistic, with values exceeding 10 considered indicative of sufficiently strong instruments. The F statistic was computed using the formula [11]:

\[ F = \frac{(N - K - 1)}{K} \times \frac{R^2}{1 - R^2} \]

MR analysis

Causal estimates were obtained through two sample MR methods, such as inverse variance weighted (IVW), weighted mode, weighted median, simple mode, and MR-Egger regression approaches. The IVW method was prioritized because it provides the most precise and statistically efficient causal estimates when all IVs are valid and horizontal pleiotropy is minimal. Under these assumptions, IVW has greater statistical power compared with other MR methods. For exposures that reached statistical significance in the IVW analysis, Bayesian weighted Mendelian randomization (BWMR) was further applied for validation [12].

Sensitivity analysis

Heterogeneity across instrumental variables was evaluated employing Q statistic, together with the respective *P*-values [13]. Potential horizontal pleiotropy was examined using the leave-one-out sensitivity test, MR-Egger intercept analyses and the MR pleiotropy residual sum and outlier (MR-PRESSO) approach [14]. In addition, scatter plots and funnel plots were generated to assess the influence of outliers and to evaluate the robustness and consistency of the MR estimates.

Statistical analysis

All statistical analyses were performed employing RStudio (version 4.5.0), and a two-sided *P*-value of less than 0.05 was considered indicative of statistical significance. For the identification of robust associations, a Bonferroni-adjusted significance threshold of *P *< 0.05/46 (approximately 1.09 × 10^-4^) was applied to account for multiple testing.

## Results

Instrumental variables

Significant SNPs were selected from the 46 exposure datasets based on predefined P-value thresholds and LD pruning criteria. SNPs with an F below 10 were removed to reduce the risk of weak IVs, and the remaining variants were carried forward for subsequent MR analyses.

MR analysis

**Figure 2:**
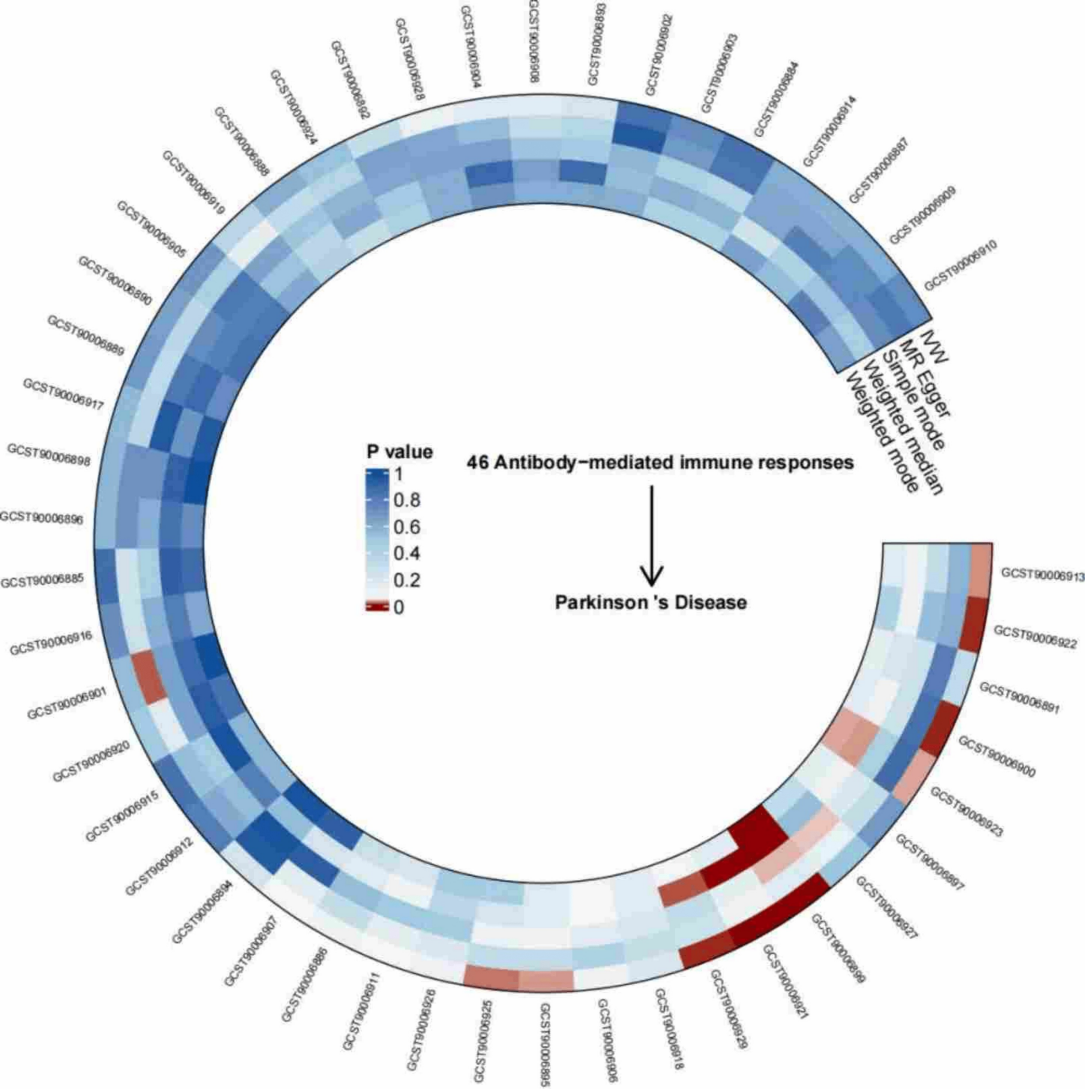
Circos heatmap depicting the causal associations between 46 antibody-mediated immune responses and PD, as determined by MR analysis PD, Parkinson’s disease; IVW, inverse variance weighted; MR, Mendelian randomization

We assessed the causal effects of 46 antibody-mediated immune responses on PD risk. After Bonferroni correction at a significance threshold of 0.05, genetically predicted levels of two antibody-mediated immune responses were significantly associated with PD risk, whereas no evidence of association was observed for the remaining 44 immune traits (Figure 2). Specifically, IVW analysis revealed that genetically predicted elevated antibody levels against EBV nuclear antigen 1 (EBNA-1) were related to a higher risk of PD (OR = 1.154, 95% CI: 1.098-1.214, *P* < 1.09 × 10^-4^), whereas genetically predicted seropositivity for anti-polyomavirus 2 IgG was protectively related to PD risk (OR = 0.897, 95% CI: 0.865-0.930, *P* < 1.09 × 10^-4^). 

By incorporating prior distributions, BWMR provides more robust causal estimates, particularly when sample sizes or the number of instrumental variables are limited. The BWMR results consistently indicated a positive association between EBNA-1 antibody levels and PD risk, as well as a protective association between anti-polyomavirus 2 IgG seropositivity and PD. The corresponding MR estimates are visualized in Table 1.

**Table 1:** Results of the two-sample MR analysis of Epstein-Barr virus EBNA-1 antibody levels and anti-polyomavirus 2 IgG seropositivity versus PD. BWMR, Bayesian weighted Mendelian randomization; MR, Mendelian randomization; OR, odds ratio; PD, Parkinson’s disease; CI, confidence interval; SNPs, single-nucleotide polymorphisms

Exposures	nSNP	Method	*P* adjust	OR (95%CI)
Epstein-Barr virus EBNA-1 antibody levels	85	MR Egger	1	1.084 (0.966-1.215)
		Weighted median	<0.001	1.179 (1.107-1.255)
		Inverse variance weighted	<0.001	1.154 (1.098-1.214)
		Simple mode	1	1.160 (1.012-1.329)
		weighted mode	<0.001	1.252 (1.143-1.372)
		BWMR	<0.001	1.141 (1.084-1.201)
Anti-polyomavirus 2 IgG seropositivity	40	MR Egger	1	0.902 (0.803-1.013)
		Weighted median	0.011	0.907 (0.861-0.956)
		Inverse variance weighted	<0.001	0.897 (0.865-0.930)
		Simple mode	1	0.908 (0.804-1.027)
		weighted mode	1	0.922 (0.825-1.031)
		BWMR	<0.001	0.891 (0.858-0.925)

Sensitivity analysis

**Figure 3:**
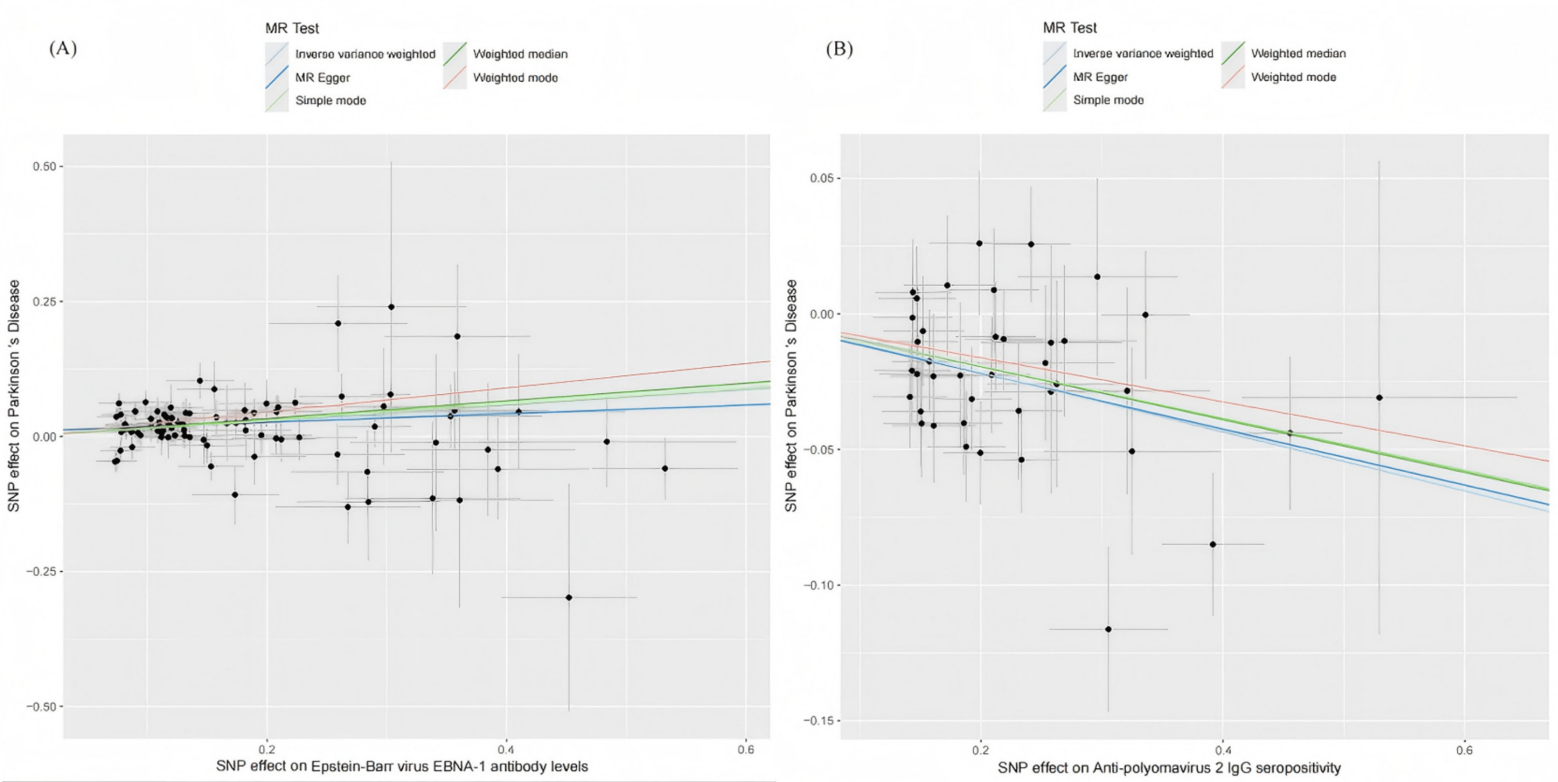
Scatter plot illustrating the two-sample MR analysis of Epstein-Barr virus EBNA-1 antibody levels and anti-polyomavirus 2 IgG seropositivity versus PD. (A) Scatter plot of the effects of Epstein-Barr virus EBNA-1 antibody levels on PD. (B) Scatter plot of the effects of anti-polyomavirus 2 IgG seropositivity on PD. MR, Mendelian randomization; PD, Parkinson’s disease

**Figure 4:**
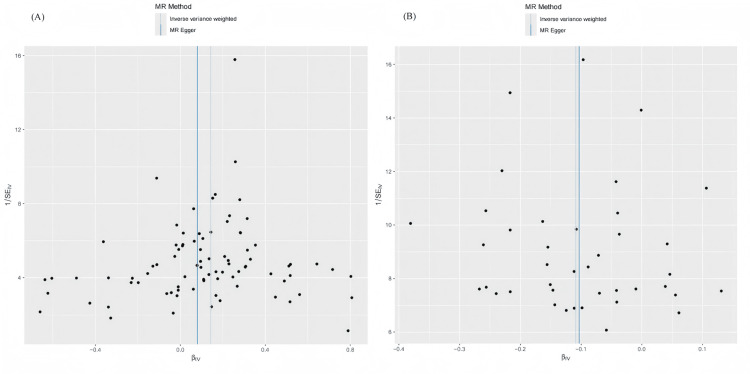
Funnel plot illustrating the two-sample MR analysis of Epstein-Barr virus EBNA-1 antibody levels and anti-polyomavirus 2 IgG seropositivity versus PD. (A) Funnel plot of the effects of Epstein-Barr virus EBNA-1 antibody levels on PD. (B) Funnel plot of the effects of anti-polyomavirus 2 IgG seropositivity on PD. MR, Mendelian randomization; PD, Parkinson’s disease

**Figure 5:**
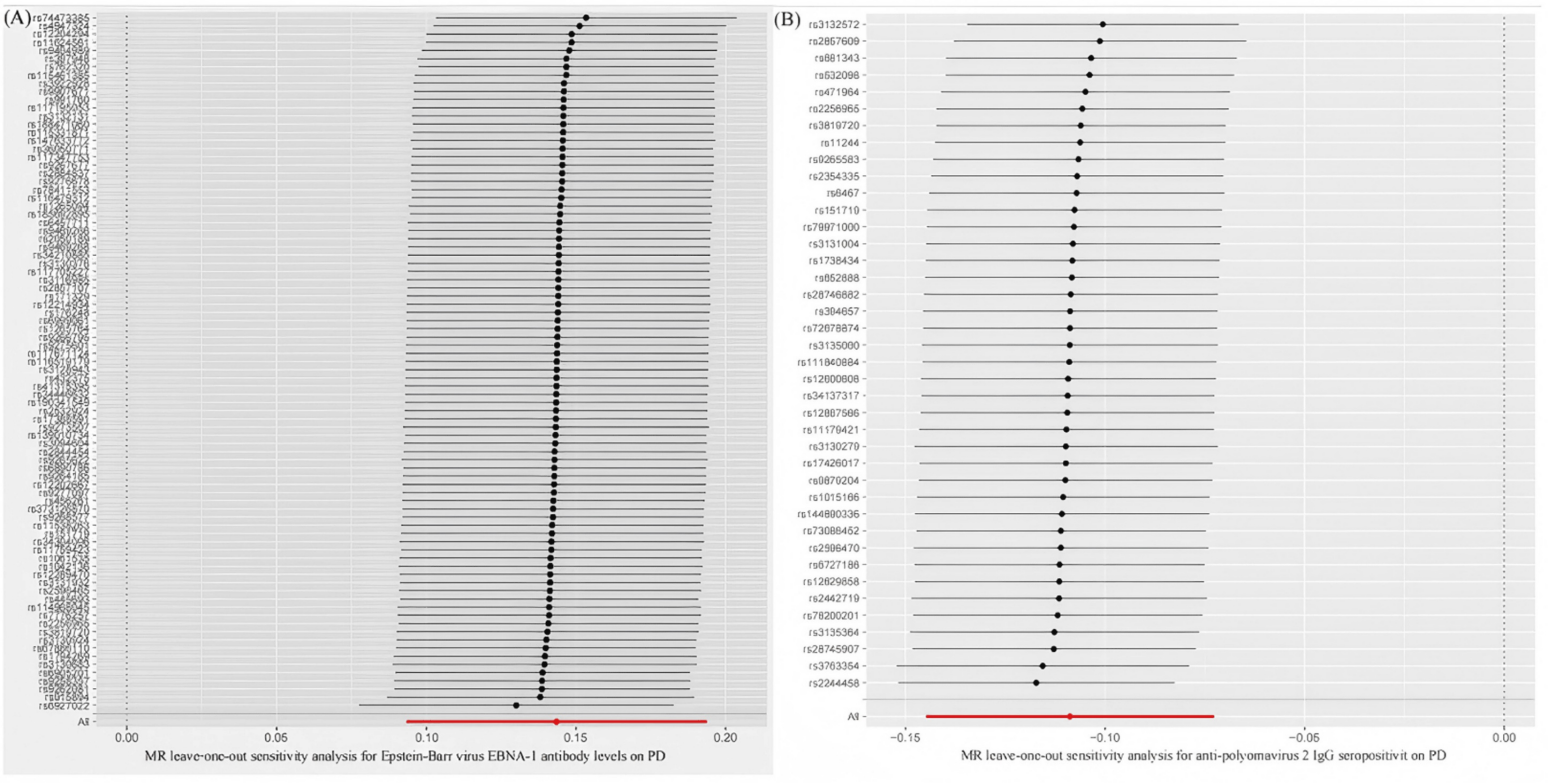
Leave-one-out plot illustrating the two-sample MR analysis of EBV EBNA-1 antibody levels and anti-polyomavirus 2 IgG seropositivity versus PD. (A) Leave-one-out analysis depicting the effects of EBV EBNA-1 antibody levels on PD. (B) Leave-one-out plot depicting the effects of anti-polyomavirus 2 IgG seropositivity on PD. MR, Mendelian randomization; PD, Parkinson’s disease; EBV, Epstein-Barr virus

To validate the stability of the above results, comprehensive sensitivity analyses were conducted. Scatter plots indicated concordant directions and magnitudes of causal estimates across all five MR methods (Figure 3). As presented in Table 2, the Q test revealed no substantial heterogeneity (*P* > 0.05), and neither the MR-PRESSO global test nor the MR-Egger intercept test detected evidence of horizontal pleiotropy or outlier SNPs (*P* > 0.05). In addition, as shown in Figures 4-5, the funnel plots showed that SNP-specific MR estimates were symmetrically distributed around the overall effect. The leave-one-out analysis did not identify any influential variants, further supporting the robustness of the results.

**Table 2:** Details of the genome-wide association studies and datasets used in our analyses. PD, Parkinson’s disease; IVW, inverse variance weighted; MR-PRESSO, MR pleiotropy residual sum and outlier

Exposures	Outcome	Heterogeneity				Pleiotropy		MR-PRESSO
		IVW		MR-Egger		Intercept	*P*-value	*P-*value
		*Q*-value	*P*-value	*Q*-value	*P*-value			
Epstein-Barr virus EBNA-1 antibody levels	PD	21.924	0.345	21.868	0.291	0.01	0.234	0.375
Anti-polyomavirus 2 IgG seropositivity		45.249	0.227	45.237	0.195	-0.001	0.92	0.233

## Discussion

Leveraging large-scale publicly available genetic data and implementing stringent SNP quality-control procedures, this study minimized potential confounding and systematically investigated the causal correlations in terms of genetic susceptibility between multiple antibody-mediated immune responses and PD. Among the 46 antibody-mediated immune responses analyzed, two exhibited significant causal effects on PD risk (*P* < 1.09 × 10^-4^). Specifically, elevated EBV EBNA-1 antibody levels were related to a higher risk of PD, whereas anti-polyomavirus 2 IgG seropositivity showed a protective association. In addition, it is worth noting that the lack of statistical significance for the two antibody-mediated immune responses in the MR-Egger analysis is more likely to reflect insufficient statistical power rather than evidence against a causal association. MR-Egger has substantially lower statistical power than IVW, particularly when the number of instrumental variables is limited. In this study, MR-Egger was primarily used as a sensitivity analysis to assess the potential impact of directional pleiotropy rather than to provide the main causal estimates. Sensitivity analyses further supported the robustness of these findings.

EBV is a large enveloped virus that was first isolated and identified in 1964 from lymphoma tissues of African children [15]. EBNA-1 is a key viral protein that is persistently expressed during latent infection and is essential for the long-term maintenance of the viral genome within host cells [16]. Previous case reports have suggested that EBV-associated encephalitis may lead to Parkinsonian syndromes, which are characterized by bradykinesia, tremor, and dystonia. This phenomenon may result from immune-mediated mechanisms or direct viral neuroinvasion affecting basal ganglia function, leading to extrapyramidal symptoms [17]. In addition, molecular modeling studies have suggested that EBV latent membrane protein 1 (LMP1) shares sequence similarities with α-synuclein, potentially triggering cross-reactive immune responses or promoting chronic neuroinflammatory pathways [18]. However, these studies primarily focused on EBV infection itself or specific viral proteins, rather than on EBNA-1 antibody levels as a distinct biomarker. To date, no observational studies have reported an association between EBV EBNA-1 antibody levels and PD. Elevated EBNA-1 antibody levels have been observed in patients with multiple sclerosis, another neurodegenerative disorder, relative to healthy controls [19]. Our study demonstrates a substantial relationship between PD and EBV EBNA-1 antibody levels, which provides a genome-wide perspective and identifies EBNA-1 antibody levels as a potential biomarker and therapeutic target for PD.

Polyomaviruses are a group of small double-stranded DNA viruses, of which more than a dozen species are known to infect humans. The most common human polyomaviruses include Merkel cell polyomavirus (MCPyV), BK virus (BKV), and John Cunningham virus (JCV) [20]. Polyomavirus-associated nephropathy is associated with active BKV infection, whereas progressive multifocal leukoencephalopathy primarily results from JCV reactivation in immunocompromised individuals [20]. Serum antibodies can indicate prior infection. Antibodies against the MCPyV T antigen are closely associated with Merkel cell carcinoma, which is significantly more prevalent in patients than in healthy controls. Moreover, changes in antibody titers can be used to monitor disease progression and the risk of recurrence [21]. To date, no studies have linked anti-polyomavirus antibodies to PD. In the present study, anti-polyomavirus antibody seropositivity was inversely associated with PD risk, although the protective effect was modest (OR ≈ 0.9). In addition, we identified seven other antibody-mediated immune responses that showed potential associations with PD; however, their associations did not remain significant after multiple-testing correction, and thus were not further explored to minimize the risk of false-positive findings. Notably, both polyomavirus 2 JC VP1 antibody levels and anti-MCPyV IgG seropositivity also exhibited protective trends, suggesting that these observations may not be coincidental. Further experimental studies are warranted to elucidate the underlying mechanisms linking polyomavirus-related immune responses to PD.

Nevertheless, several limitations of the present study warrant careful consideration. First, the GWAS datasets for antibody-mediated immune responses and PD were derived from independent cohorts. This may have introduced heterogeneity in sample size, study design, and quality control procedures. Second, all analyzed GWAS data were limited to individuals of European ancestry, which restricts the generalizability of our findings to other ethnic populations. Finally, although we identified potential causal associations between 46 antibody-mediated immune responses and PD, the underlying biological mechanisms remain largely unexplored. These mechanisms warrant further experimental and mechanistic investigation.

## Conclusions

By leveraging high-throughput GWAS datasets, we systematically examined, for the first time, the relationships between 46 pathogen-related antibody-mediated immune responses and PD risk. Our findings indicate a causal association between genetically elevated EBV EBNA-1 antibody levels and an increased risk of PD, whereas genetic susceptibility to seropositivity for anti-polyomavirus 2 IgG appears to be associated with a reduced risk. From a clinical perspective, these results suggest that antibody-mediated immune profiles may contribute to individual differences in PD susceptibility and could inform future risk stratification strategies. Moreover, the identified immune-related pathways may help prioritize candidate biomarkers for early disease risk assessment and guide the development of immunomodulatory or antiviral therapeutic approaches, pending further experimental validation. Collectively, these findings provide mechanistic insights into PD etiology while offering a framework for future translational and clinical research.
